# Prioritization of carceral spending in U.S. cities: Development of the Carceral Resource Index (CRI) and the role of race and income inequality

**DOI:** 10.1371/journal.pone.0276818

**Published:** 2022-12-15

**Authors:** Britt Skaathun, Francesca Maviglia, Anh Vo, Allison McBride, Sarah Seymour, Sebastian Mendez, Gregg Gonsalves, Leo Beletsky

**Affiliations:** 1 Division of Infectious Diseases and Global Public Health, University of California San Diego, San Diego, CA, United States of America; 2 Yale School of Public Health, Public Health Modeling Unit, Yale University, New Haven, CT, United States of America; 3 Health in Justice Action Lab, School of Law and Bouvé College of Health Sciences, Northeastern University, Boston, MA, United States of America; University of North Carolina at Chapel Hill, UNITED STATES

## Abstract

**Background:**

Policing, corrections, and other carceral institutions are under scrutiny for driving health harms, while receiving disproportionate resources at the expense of prevention and other services. Amidst renewed interest in structural determinants of health, roles of race and class in shaping government investment priorities are poorly understood.

**Methods:**

Based on the Social Conflict Model, we assessed relationships between city racial/ economic profiles measured by the Index of Concentration at the Extremes (ICE) and budgetary priorities measured by the novel Carceral Resource Index (CRI), contrasting investments in carceral systems with funding for health and social support across the 50 most populous cities in the United States (U.S.). Bivariate correlations, and unadjusted and adjusted polynomial regression models were used to assess the relationship between budgetary investments and population concentration at extremes in terms of income, racial/ethnic composition, and education, controlling for other demographic characteristics.

**Results:**

In our sample, median CRI was -0.59 (IQR -0.64, -0.45), with only seven cities exhibiting positive CRI values. This indicates that most large U.S. cities spend more on carceral systems than on health and supportive services, combined. Adjusted polynomial models showed a convex relationship between the CRI and ICE-Education, and ICE-Race(White vs. Black)+Income, with quadratic terms that were positive and significant at p<0.05. After controlling for age, the strongest prioritization of carceral systems was observed in cities where the proportion of low-income Black residents approached or exceeded that of high-income white residents.

**Conclusions:**

Municipal prioritization of carceral investments over health and social support is pervasive in the U.S and exacerbated by racial and economic disparities. The CRI offers new opportunities to understand the role of government investments as a structural determinant of health and safety. Longitudinal research is warranted to examine the relationship between budget priorities, structural racism, and health outcomes.

## Introduction

Since June of 2020, following the death of George Floyd, there has been growing pressure on governments to divest from the funding of police, and reinvest these funds in the health and wellbeing of communities, particularly communities of color [1, 2]. However, municipalities currently lack a quantitative metric that jointly considers investment in carceral, health, and social systems. We introduce a new metric, the Carceral Resource Index (CRI), that can be used to evaluate municipal priorities by contrasting investments in systems of punishment and control relative to spending on health and supportive services. The goal of this analysis is to demonstrate the utility of the CRI by testing whether the social conflict model informs fiscal priorities.

There is increased recognition that policing and carceral systems have received disproportionate fiscal resources; this has crowded out the funding of health and social initiatives and led to health harms. Heavy investment in policing and other carceral systems has been linked to homelessness, substance use, poor birth outcomes, nonfatal injury, anxiety, posttraumatic stress disorder, and death [3, 4]https://www.zotero.org/google-docs/?tqOGoo, whereas investment in public services, housing, education, and the environment has been consistently linked to positive physical and mental health outcomes, including decreased homicide rates [5–7].

Despite this evidence, total spending on carceral systems in the United States (U.S.) has increased by 382% after accounting for inflation across federal, state, and local levels between 1980 and 2018, going from 29.6 billion dollars in 1980 to 344.6 billion dollars in 2018 [8]. In contrast, spending on cash welfare programs—including Temporary Assistance for Needy Families (TANF), food stamps, and supplemental Social Security payments—only increased by half as much [8]. As a result, the U.S. currently spends more than twice as much on carceral systems than on welfare.

### What might explain the heavy investment in carceral systems?

Two main explanatory models have been proposed for the allocation of resources to policing. The first, known as the public demand model, posits that resource allocation is determined by demands for public safety in response to fluctuations in crime rates [9]. The second, known as the social conflict model, understands policing as a tool of social control that responds to the interests of social elites, whether economic or racial [10]. Proponents of the social conflict model explain resource allocation to police as a response to racial and class threats, whereby White and/or wealthy residents demand increased policing as the number and proximity of non-White and/or poor residents increases. Consistent with this model, a recent analysis of national public health survey data demonstrated that white respondents who exhibited racial resentment were less likely to support the decriminalization of drug possession [11].

There is little support for the public demand model, with several studies finding no relationship between rates of violent crime and police spending [12–14]. Police and carceral spending have continued to significantly increase despite crime rates declining since the 1990s [14, 15]. On the other hand, there is growing evidence in support of the social conflict model. The size or funding of the police force has been linked to the growth in the proportion of the Black population [14–16], the Hispanic and Black population [16–19], and the level of racial segregation in a given area [20]. Similarly, economic inequality and wealth segregation were shown to be significantly associated with increased resource allocation to policing [12, 21]. However, some studies did not find support for this hypothesis [17, 18].

### Updated analyses are needed

The impact of racial and class threats on carceral structures has not been widely examined since prior to the 21st century, and those that have largely ignored possible interactions between municipal racial composition and wealth distribution [12, 14, 17, 18, 22].

Most importantly, previous literature has treated determinants of police funding and correctional spending, and spending on health, welfare, and social services separately, when there is evidence that these three funding streams are not independent. As evidenced by Saez and Zucman (2019), the increase in spending on carceral systems occurred in parallel to a decrease in spending on welfare in the U.S. Likewise, the interdependency between these funding streams has been exemplified through the impact of Medicaid expansion on recidivism. In a comparative interrupted time series analysis between six large urban U.S. counties, Medicaid expansion reduced both the probability of rearrest and the number of arrests in two of the three county pairs assessed [23]. Therefore, a metric that considers these two budgetary items jointly is warranted.

### The current study

The goal of this analysis is to introduce a new metric, the CRI, (which is designed to measure a local government’s fiscal commitment to carceral systems) and test its utility. The CRI captures the municipal prioritization of carceral versus health and social support systems; thus, this study makes an innovative contribution to the literature by jointly examining these different funding streams. Our preliminary analyses using the CRI demonstrate a positive correlation between cities that prioritize carceral spending and cities with large Black/African-American populations [24]. This study builds on these initial insights by employing more complex indices that measure racial and economic distribution combined. To our knowledge, this is the first study to consider the joint interaction of race and class on the prioritization of carceral systems over health and social support systems. We examine the utility of the CRI by quantifying the relationship between racial and economic equity and budgetary investment priorities across major U.S. cities.

## Methods

### Sample

**Fig 1:**
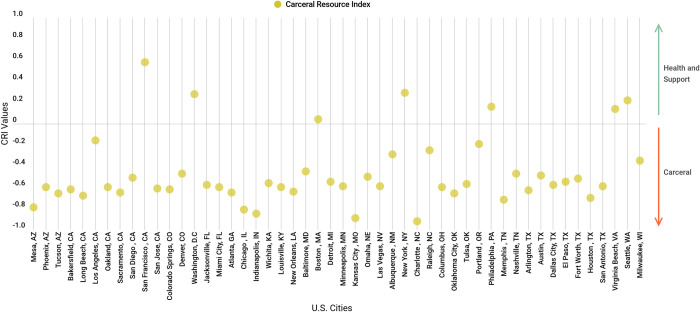
Carceral Resource Index by U.S cities (n = 50 cities).

We conducted our analysis on the 50 most populated cities in the U.S. according to the most recent census data. We calculated a CRI value, described below, for all cities in the year 2017. Two cities (Detroit, Michigan and Oakland, California) did not have publicly available adopted 2017 budgets. In these cases, 2016 budgets were substituted and converted to 2017 dollars, accounting for inflation. In addition, the 2016–17 budget for Fresno, California and Tampa, Florida were not suited for analysis as they did not allow for a breakdown of departments. Therefore, the 51st and 52nd most populated cities were included instead: New Orleans, Louisiana and Wichita, Kansas. Our final sample consisted of 50 cities spanning 30 states. A list of these cities can be found in **Fig 1**.

### Variables

#### Dependent variable

Our dependent variable of interest is the Carceral Resource Index (CRI) developed by the Health in Justice Action Lab at the Northeastern University School of Law. The CRI is designed to measure a government’s fiscal commitment to carceral systems [24]. This metric contrasts investments in systems of punishment and control relative to spending on health and supportive services. As inputs, the CRI captures government budgetary outlays in three categories: carceral, health, and social support. Using data from the adopted budgets in each city, the CRI is therefore calculated as: [(health + support)—carceral]/total budget].

CRI values range from -1 to 1. A CRI coefficient of -1 represents a jurisdiction’s total fiscal prioritization of carceral systems to the exclusion of health and support, while 1 represents total fiscal prioritization of health and support systems to the exclusion of carceral expenditures. Actual composition of budget outlays in each CRI category will vary by local government design. In this way, the CRI is an indicator of a city’s investment priorities, rather than an exact assessment of spending within a city. Though the social conflict model explored in this paper concerns itself with the allocation of resources to the police, the benefit of also including health and social costs in developing this index—as opposed to solely quantifying each city’s reliance on carceral systems—is that it attenuates the risk of establishing false equivalencies between cities that are purely carceral versus those with more egalitarian priorities.

*CRI Inclusion & exclusion parameters*. Departments or services were included if they had a direct association with overall population health or with criminal justice. They were excluded if they fell between both carceral and health/support systems, or did not mediate carceral and health/support outcomes. Government budgetary outlays measured by the CRI are specific to local-level appropriations, which excludes county/state/federal funding. Below we note the main departments included in each spending category; an extended description of the methodology is available on the CRI portal [24].

*Carceral*. The following departments and agencies comprise the carceral spending category: police departments, corrections, prosecutors and public defense, courts, sheriff’s offices, probation and community supervision.

While public defenders’ offices and court-appointed attorneys serve a vital role for people accused of all manner of crimes, we included such spending in the carceral category given that it is situated within the overall spending for courts and law enforcement under city budgets. In some city budgets, public defenders did not have a line item under court and law enforcement spending, so we could not assume that percentage of allocation for public defenders in these cities. In order to keep analyses consistent in measuring carceral spending, we situated public defenders under the carceral category.

*Health*. The health spending category includes the following departments: health and human services, public health, public spaces, parks and recreation. We included the latter two departments in the health spending category because access to public space and investments in social infrastructure have been shown to promote community health.

We excluded the offices of chief medical examiners because their roles often involve working with police investigators on potentially crime-related deaths; in some counties, the sheriff is also the coroner.

Environmental protection was temporarily excluded due to challenges in identifying what was being funded. Environmental and climate protection spending clearly improves public health and may reduce the fear of crime [25]. Additionally, interventions which seek to reduce air pollution, the presence of heat islands, and lead pollution could reduce crime and violence [26–28]. However, outside of unusual jurisdictions like the District of Columbia, relatively little environmental spending comes from the municipal level. In addition, it is challenging to disaggregate which agency is responsible for related initiatives, and it is difficult to distinguish the direct impact of environmental design from the beneficial effects of the organizational development surrounding such environmental efforts [29].

*Support services*. The support services category included the following departments: housing, neighborhood development, employment, community engagement, arts and culture. We included Arts and Culture departments because qualitative studies have shown engagement in the arts can promote active citizenship, self-esteem, and strengthen community ties [30].

We excluded child and family services departments because, in addition to the human services they provide, they are also often related with Child Protective Services (CPS) divisions and programs supporting juvenile detention initiatives. Child and family services have been widely criticized for deploying service modalities with carceral and racist foundations, including war on drugs policies used to separate families and route children into foster care [31].

Similarly, we excluded education from this category because the relationship between education and carceral systems is blurred by the potential use of education dollars in support of carceral expenditures. As we have discovered, this could include school resource officers and criminal justice curriculum, in which schools are preparing young adults to enter in the field of corrections or law enforcement [32]. In order to include education, deeper investigations would need to be conducted into if schools within each city are providing this type of curriculum or if they employ resource officers.

#### Independent variables

*Index of Concentration at the Extremes (ICE)*. The Index of Concentration at the Extremes (ICE) is a measure of the extent to which a population is concentrated into the highest and lowest extreme. It was developed by Douglas Massey in 2001 and first introduced for the purpose of monitoring population health by Nancy Krieger in 2016 [33, 34]. Its values range from -1 (representing extreme deprivation) to 1 (representing extreme privilege). It is calculated using public data from the American Community Survey (ACS) 5-year estimates from the US Census Bureau [35]. The original ICE formula, as developed by Massey, is as follows: A is the number of affluent persons in neighborhood *i*, *P*_*i*_ the number of poor persons in neighborhood *i* and *T*_*i*_ the total population for whom income level is known in neighborhood *i*; *ICE*_*i*_ *= (A*_*i*_*-P*_*i*_*)/T*_*i*_ [33]. The ICE has traditionally been calculated at the neighborhood level, but can be calculated at any geographic level. Following the work of Feldman et al. [36], we calculate the ICE at the city level and jointly assess extreme concentrations of both income and racial composition, education, and homeownership as described in **Table 1** below.

**Table 1:** Calculation of the Index of Concentration at the Extreme (ICE) variables (using American Community Survey (ACS) 5-year estimates, 2013–2017), adopted from Feldman et al (2015).

Domain	Variable name	Formula	ACS Table ID
Income	ICE_income_	[(over US$100 k)–(under US$25 k)]/total population_household income	B19001
Education	ICE_education_	[(4 years college or more)–(less than high school)]/total population_education. *Note*: *educational level determined solely for adults ≥25 years old*	B15002
Race/ethnicity	ICE_race_wb_	[(white non-Hispanic)–(black non-Hispanic)]/total population_race	B03002
Income and race/ethnicity combined	ICE_wb+income_	[(white non-Hispanic over US$100 k)–(black alone under US$25 k)]/total population_household income	B19001
Income and race/ ethnicity combined	ICE_wpc+income_	[(white non-Hispanic over US$100 k)–(total under US$25 k–white non-hispanic under US$25 k)]/total population_household income	B19001
Homeownership and race/ethnicity combined	ICE_wb+homeownership_	[(white non-Hispanic owner-occupied housing units)—(black alone renter-occupied housing units)]/total occupied housing units	S2502
Homeownership and race/ethnicity combined	ICE_wpc+homeownership_	[(white non-Hispanic owner-occupied housing units)—(total renter-occupied housing units—white non-hispanic renter-occupied housing units)]/total occupied housing units	S2502

#### Covariates

*Age*, *income*, *and race*. Spending for public education, public safety, and recreational services are likely to be affected by the age distribution of the population, with elderly citizens typically requiring higher health expenditures and more attention by social services; therefore, the age distribution within each city was controlled for in the analyses. Similarly, higher-income households and racially privileged groups often demand more services from the government. Income was controlled for in models where the independent variable does not already include some measure of income (e.g. when testing the *ICE-Education* variable, but not when testing the *ICE-Income* variable). Race/ethnicity was controlled for in models where it was not already captured by the ICE index. Age, race/ethnicity, and income data were obtained from the US census via the American Community Survey (ACS Table ID: B19001, S0101). We calculated the proportion of people under 18 years old, people 65 years or older, people earning less than 25K/year and people earning more than 100K/year, and the proportion of the population that is non-Hispanic White within each of the corresponding cities included in this analysis.

*Crime*. According to the public demand model, increases in violent and property crime prompt public demands for safety, resulting in greater allocation of resources to police and other carceral systems. To verify this hypothesis, we tested the association between violent crime rate per 100,000, property crime rate per 100,000, and the CRI. Violent crime includes murder and manslaughter, rape, robbery, and aggravated assault. Property crime includes burglary, larceny-theft, motor vehicle theft, and arson. Given that the CRI is calculated for the year 2017, we considered crime rates in 2016 under the assumption that public demand would influence the budget for the following year. Violent and property crime rates were calculated based on the data from the FBI Uniform Crime Reporting (UCR); since data concerning Raleigh, North Carolina was missing from the UCR platform, the city was excluded from the crime analysis [37].

### Statistical analysis

**Fig 2:**
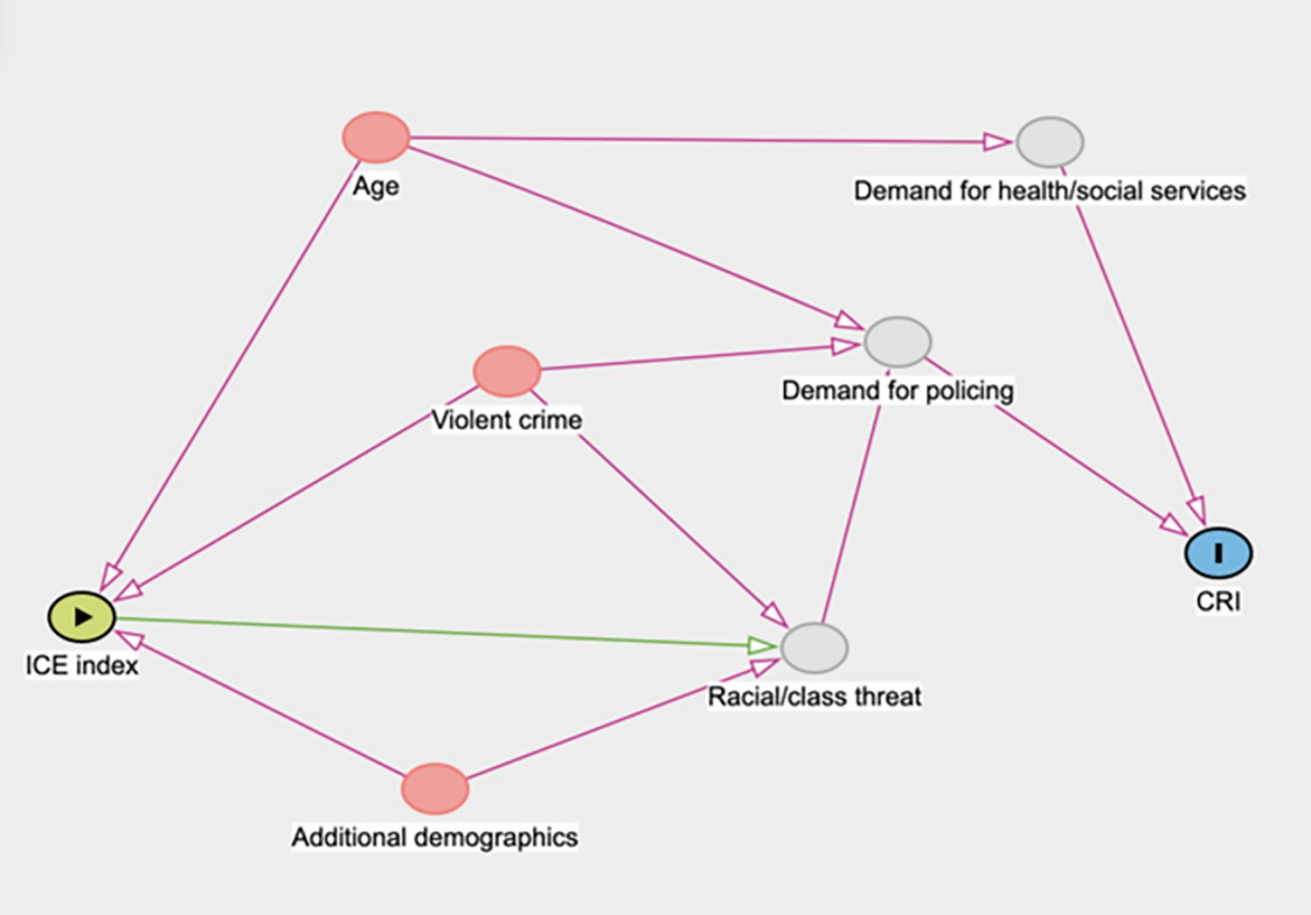
Directed acyclic graph (DAG) showing the theoretical model informing analysis. The green variable represents the exposure variable; the blue variable represents the outcome variable; grey variables represent unobserved variables; pink variables represent confounders; pink lines show biasing paths; green lines show causal paths. Additional demographics included proportion of high income earners, proportion of elderly residents, or proportion of non-Hispanic White residents depending on the model. Bivariate associations between each covariate and the CRI were assessed with Pearson correlations when continuous and Gaussian (Proportion of population ≥ 65 yrs of age, Property Crime per 100,000 in 2016, ICE_income,_ ICE_education)_ and with a Spearman’s rank correlation when the variables were not Gaussian. Some of the covariates presented in this model were not found to be significantly associated with the CRI and were thus not included in the adjusted models.

Our analyses were guided by the directed acyclic graph (DAG) represented in **Fig 2**. Unadjusted and adjusted linear and polynomial regression models were used to assess the relationship between the CRI and city characteristics represented by seven Indices of Concentration at the Extremes.

We opted to use a polynomial regression model over a linear regression model for several reasons. First, Jacobs & Helms [21] found a U-shaped relationship between economic inequality and the size of the police force, and D’Alessio, Eitle, & Stolzenberg [18] obtained a similar result when examining private police: thus, we expected that a polynomial regression may better describe the relationship between the ICE variables and the CRI. Additionally, after examining the diagnostics of the residuals of the linear models comparing the residuals vs. fitted plots (**S1 Fig**) and the bivariate scatter plots (**S2 Fig**), curvilinear relationships between the ICE variables and the CRI indicated that polynomial regression models were better fit to the data. For ease of interpretation, the ICE variables were standardized using a z-transformation.

ICE indicators with large magnitudes of association in unadjusted models were considered in separate adjusted models to estimate the total effect of each indicator on CRI [38]. Potential confounding variables were selected based on *a priori* knowledge about their interrelationships with the exposures of interest and spending priorities as depicted in the DAG and mentioned above. They were assessed in bivariate analyses with the CRI, and selected for inclusion in adjusted models based on their magnitude of association. Finally, heteroscedasticity and normality of the models’ residuals were checked via examination of the selected models’ residuals vs. fitted plots (**S3 Fig**) and normal probability plots (**S4 Fig**), which were found to adequately meet both assumptions prerequisite for this modeling approach.

All regression analyses were conducted using R statistical computing language, version 4.1.3 [39].

## Results

### CRI distribution

The median CRI value across the 50 cities in 2017 was -0.59 (IQR -0.64, -0.45), indicating a strong preference towards funding carceral resources over health and social support **(Fig 2)**. **Table 2** details summary characteristics between CRI values and measures of sociodemographic distributions within our cities sample. The five cities that most strongly favored carceral spending over health and social support were: Charlotte, North Carolina (CRI -0.92), Kansas City, Missouri (CRI -0.89), Indianapolis, Indiana (CRI -0.85), Chicago, Illinois (CRI -0,81), and Mesa, Arizona (-0.79). The five cities that most strongly favored health and social support over carceral spending were: San Francisco, California (CRI 0.58), New York, New York (CRI 0.29), Washington, D.C. (CRI 0.28), Seattle, Washington (CRI 0.22), and Philadelphia, Pennsylvania (0.16) **(Fig 2)**.

**Table 2:** Summary characteristics between CRI values and cities’ demographic distributions.

	Cities with CRI Equal or Below Sample Median (N = 25)	Cities with CRI Above Sample Median (N = 25)	All Cities (N = 50)
**City population in 2020 (thousand of people)**			
Mean (SD)	831 (612)	1200 (1650)	1020 (1250)
Median [Min, Max]	554 [390, 2690]	710 [389, 8320]	663 [389, 8320]
**Proportion of population age <18**			
Mean (SD)	0.133 (0.0202)	0.136 (0.0301)	0.135 (0.0254)
Median [Min, Max]	0.135 [0.103, 0.177]	0.134 [0.0954, 0.252]	0.134 [0.0954, 0.252]
**Proportion of population age ≥ 65**			
Mean (SD)	0.226 (0.0281)	0.208 (0.0379)	0.217 (0.0342)
Median [Min, Max]	0.225 [0.170, 0.298]	0.206 [0.134, 0.270]	0.219 [0.134, 0.298]
**Proportion of population with income <$25K**			
Mean (SD)	0.249 (0.0571)	0.238 (0.0706)	0.243 (0.0638)
Median [Min, Max]	0.247 [0.127, 0.388]	0.219 [0.124, 0.461]	0.233 [0.124, 0.461]
**Proportion of population with income >$100K**			
Mean (SD)	0.230 (0.0710)	0.258 (0.0939)	0.244 (0.0836)
Median [Min, Max]	0.220 [0.123, 0.486]	0.245 [0.0718, 0.488]	0.233 [0.0718, 0.488]
**Proportion of Non-Hispanic Whites in population**			
Mean (SD)	0.402 (0.149)	0.444 (0.173)	0.423 (0.161)
Median [Min, Max]	0.392 [0.133, 0.673]	0.426 [0.106, 0.835]	0.402 [0.106, 0.835]
**Violent crime rate per 100,000 (2016)**			
Mean (SD)	878 (381)	839 (457)	859 (416)
Median [Min, Max]	783 [373, 1820]	709 [155, 2050]	719 [155, 2050]
**Property crime rate per 100,000 (2016)**			
Mean (SD)	4070 (984)	3850 (1430)	3960 (1210)
Median [Min, Max]	4100 [2340, 5860]	3640 [1460, 6860]	3920 [1460, 6860]

### ICE distribution

The median and interquartile range for the ICE indicators were as follows: ICE-Income: -0.003 (-0.055, 0.068),_,_ ICE-Education: 0.175 (0.099, 0.315), ICE-Race(White vs. Black): 0.228 (0.049, 0.396), ICE-Race(White vs. Black)+Income: 0.095 (0.038, 0.150), ICE-Race(White vs. People of Color)+Income: 0.017 (-0.038, 0.091), ICE-Race(White vs. Black)+Homeownership: 0.193 (0.026, 0.269), and ICE-Race(White vs. People of Color)+Homeownership: 0.022 (-0.142, 0.145). Detroit, Michigan had the lowest ICE score in every category except education, where it had the second to lowest score (and was surpassed by Milwaukee, Wisconsin), and homeownership (where Minneapolis, Minnesota had the lowest score). In contrast, Seattle, Washington had the highest score for ICE-Education,_,_ ICE-Race(White vs. Black)+Income,_,_ and ICE-Race(White vs. People of Color)+Income._,_ Portland, Oregon had the highest score for ICE-Race(White vs. Black);_,_ San Jose, California had the highest score for ICE-Income; Mesa, Arizona had the highest score for ICE-Race(White vs. Black)+Homeownership; and Colorado Springs, Colorado had the highest score for ICE-Race(White vs. People of Color)+Homeownership.

### Covariates

**Fig 3:**
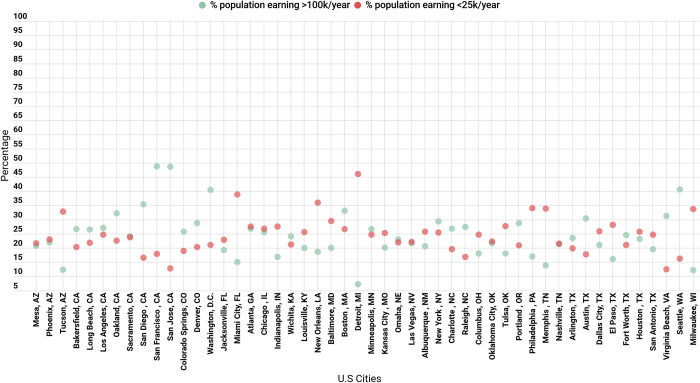
Income distribution by U.S cities (n = 50 cities).

**Fig 4:**
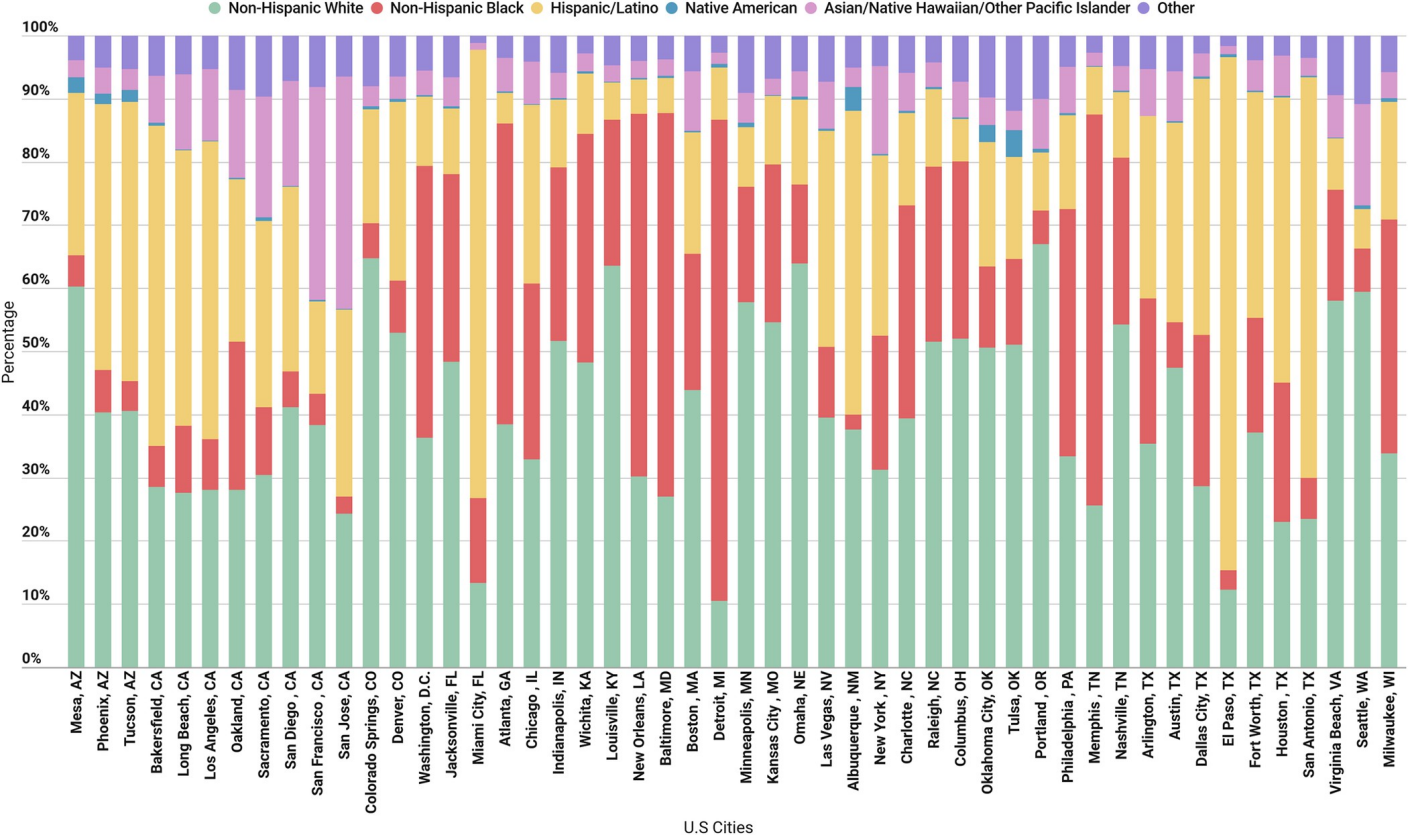
Race and ethnicity distribution by U.S cities, (n = 50 cities).

The proportion of the population earning less than $25K/year and the proportion of the population earning more than $100K/year can be seen in **Fig 3**. Detroit, Michigan has the lowest proportion of the population earning more than $100k/year at 7%, and the largest proportion of the population earning less than $25K/year at 46%. In contrast, Nashville, Tennessee has the most balanced income distribution, with approximately 21% of the population earning more than $100K/year, and 21% earning less than $25K/year **(Fig 3)**. Bakersfield, California had the oldest population, with 30% over the age of 64, and Wichita, Kansas had the youngest, with 25% under the age of 18. Wichita, Kansas had the population with the largest proportion of non-Hispanic, White residents (83%); (**Fig 4**). Violent and property crime were calculated for the 49 cities in our sample with available data (no property or crime rate data were available for Raleigh, NC). The violent crime rate was highest in Detroit, Michigan with 2046.5 incidents per 100,000 residents and lowest in Virginia Beach, Virginia with 154.5 incidents per 100,000 residents.

### Statistical model results

The bivariate associations between each independent variable, covariate, and CRI can be found in **Table 3** below.

**Table 3:** Bivariate analyses of independent variables with the carceral resource index, United States (n = 50 cities).

	Spearman’s Rho Coefficient	p-value
Proportion of population < 18 yrs of age	0.0223	0.876
Proportion of population ≥ 65 yrs of age	-0.5002	**0.002**
Proportion of population income < $25k	-0.1619	0.246
Proportion of population income > $100k	0.5120	**0.016**
Proportion non-Hispanic, White	0.0604	0.677
Violent Crime per 100,000 in 2016	-0.1619	0.261
Property Crime per 100,000 in 2016	-0.1692	0.235
ICE_income_	0.3980	**0.0042**
ICE_education_	0.3987	**0.0041**
ICE_race_wb_	0.1190	0.406
ICE_wb+income_	0.3483	**0.012**
ICE_wpc+income_	0.2659	0.059
ICE_wb+homeownership_	0.0486	0.734
ICE_wpc+homeownership_	0.0090	0.951

The CRI was positively correlated with the proportion of people earning more than $100K/year (coefficient = 0.5120,p = 0.001) and negatively correlated with the proportion of people 65 years of age or older (coefficient = -0.5002, p = 0.002). The CRI was not significantly correlated with the proportion of people under 18 years of age, the proportion of people earning less than $25K/year, the proportion of the population that is non-Hispanic White, or the rate of violent crime per 100,000 residents. All of the ICE indices with the exception of race and homeownership were significantly and positively correlated with the CRI at p<0.05 in the bivariate analyses.

The diagnostics of the residuals of the linear models comparing the residuals to the fitted plots depicted a curvilinear relationship between the CRI and ICE-Income, ICE-Education, ICE-Race(White vs. Black)+Income,_,_ and ICE-Race(White vs. People of Color)+Income. These relationships were assessed using polynomial regression models, adjusting for covariates specific to each model (informed by the DAG). Results from adjusted linear regression version of selected models are also provided in **S1 Table** for purposes of robustness assessment and comparison check against their polynomial model counterparts.

**Fig 5:**
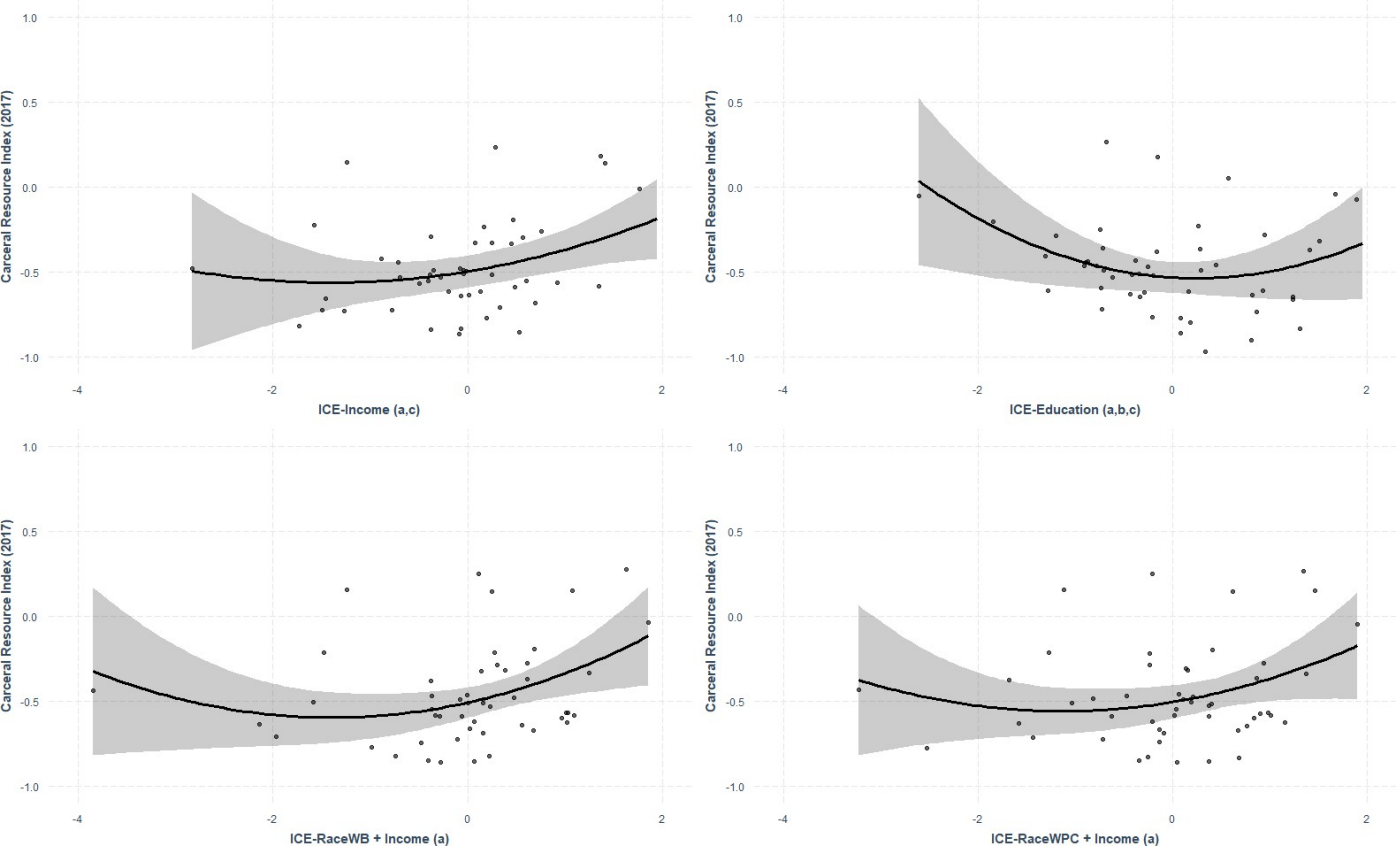
Partial residual plots of the relationships between the CRI and ICE variables, controlling for confounders (n = 50 cities). All ICE variables’ values shown are standardized and scaled by z-transformations. ICE-Income adjusts for proportion of population ≥ 65 yrs of age and the proportion of population non-Hispanic White. ICE-Education adjusts for proportion of population ≥ 65 yrs of age and the proportion of population non-Hispanic White, and the proportion of population income ≥ $100k, ICE-RaceWB and ICE-RaceWPC adjust for all three.

Model 1 was adjusted for the proportion of the population ≥ 65 years of age and the proportion of the population non-Hispanic White, Model 2 was adjusted for the proportion of population ≥ 65 years of age and the proportion of population non-Hispanic White, and the proportion of population income ≥ $100k, and models 3 and 4 adjust for the proportion of the population ≥ 65 years of age. The adjusted polynomial models showed that the quadratic term was positive and significant between the ICE measures and the CRI after adjusting for potential confounders in the models assessing CRI and ICE-Education and ICE-Race(White vs. Black)+Income. This indicates a convex relationship, where fiscal prioritization favors health and social support in instances where the population is Black and low-income (or with less education) or White and high-income (or with more education), represented by the tails of the ICE variable values. Fiscal prioritization favors carceral spending when ICE values indicate that the proportion of privileged and deprived populations are more balanced (**Table 4, Fig 5**). While the linear relationship between ICE-Income and CRI was significant, the quadratic term was not significant in the polynomial model after adjusting for confounders.

**Table 4:** Adjusted polynomial regression analyses: Factors associated with the carceral resource index, United States (n = 50 cities).

**Model 1**	Coefficient	Standard Error	p-value	Adjusted R^2^
ICE_income_	0.6570	0.3084	**0.0386**	0.290
(ICE_income_)^2	0.3977	0.3112	0.2077	
**Model 2**				
ICE_education_	-0.1595	0.5018.	0.7521	0.377
(ICE_education_)^2	0.7066	0.2677	**0.0115**	
**Model 3**				
ICE_wb+income_	0.4690	0.2903	0.1129	0.298
(ICE_wb+income_)^2	0.5849	0.2862	**0.0468**	
**Model 4**				
ICE_wpc+income_	0.3969	0.2987	0.1905	0.261
(ICE_wpc+income_)^2	0.4536	0.2986	0.1355	

## Discussion

This study introduced a new metric, the CRI, to better quantify municipal budget priorities. Our findings show that the majority of the cities in our sample exhibited a negative CRI, which is indicative of prioritization of carceral systems over welfare in the U.S.; a trend that has been observed since the 1990s [8]. Only 7 cities presented a positive CRI value, and on average carceral spending was nearly 60% higher than spending on health and social support.

Consistent with previous literature, we demonstrate lack of support for the public demand model, as violent and property crime rates were not significantly associated with budget priorities for the following year [12–14].

Instead, when considering the relative proportion of privileged and underprivileged groups together, we found that health and social support spending decreased as the proportion of residents in the underprivileged group increased. For instance, in all the cities with a positive CRI value, the number of high-income White residents significantly exceeded that of low-income Black residents. Similarly, none of the cities where the proportion of low-income residents was higher than that of high-income residents had a positive CRI.

Our results are consistent with the finding of Jacob & Helms [21] that the presence of relative disparities between high- and low-income groups drives carceral investments more than absolute economic deprivation does on its own. In addition, we found that fiscal prioritization favors health and social support in instances where the population has larger proportions of people with little education (less than a high school degree) or with more education (four years of college or more), and that fiscal prioritization favors carceral spending when the proportion of the population is more educationally balanced.

This observed dynamic seems to support the class threat hypothesis, according to which higher socio-economic status (SES) residents demand an increase in policing to protect their economic interests as they feel threatened by the presence and proximity of SES residents.

Contrary to some previous studies that found the percentage of the Black population to be the strongest predictor of increased correctional and policing investments or increase in the size of the police force [14, 16–18], we found the proportion of Black and White residents out of the total population to be insignificant without accounting for income, indicating that race does not have an independent effect on investment priorities in our sample.

We observed a significant association when race and class disparities were considered jointly. Cities only prioritized health and social support when high income White residents strongly outnumbered low-income Black residents. When the proportion of these two groups was more balanced, or when low-income Black residents outnumbered high income White residents, we observed a shift towards carceral funding. A similar dynamic was observed when comparing high-income White residents and low-income residents of color, but this became insignificant after controlling for the proportion of elderly residents. These findings suggest an interaction between race and class that is aligned with the social conflict model, indicating that high income White residents may feel threatened by the presence of low-income Black residents.

Our study has some key limitations. First, the CRI focuses on municipal budgets, and therefore does not capture other forms of investments in carceral and social support systems that may come from state or federal sources. For instance, the majority of correctional spending typically comes from state governments, and local spending only accounts for one third of total correctional spending [40]. Similarly, a portion of funding for health and hospitals typically comes from state sources [41]. Additionally, the structure of municipal budgets often masks the total actual spending on policing since overtime wages for police officers are typically included in a broader overtime wages line item that includes overtime wages paid by other departments as well; hence, direct expenditures on police departments tend to underestimate actual spending on police. Therefore, the CRI should not be taken to represent the entirety of investments in carceral and social support systems in a given geographical area, but rather as an indicator of the approach prioritized by local governments. Other barriers which distort the precision of the CRI as a tool for tracking appropriations arise due to inconsistencies in how each of the cities in our sample chose to prepare their budget. We believe that future researchers would benefit from a consistent approach which prioritizes a standardized, easy-to-navigate budget document amenable to the application of modern algorithms. Given the tedious nature of manually coding each budget document by hand, enabling computer programs to parse out financial and categorical data of interest has the potential to enhance the quality and quantity of further socio-epidemiological research concerning the influence of economic appropriations.

Moreover, the CRI at this stage does not consider the partisan context of each city’s budgets. Questions remain as to how political affiliation can be operationalized given the different branches of government, the separate weight of their functions, and the inter-party variety seen in American political thought. That being said, this lack of exploration does not undermine the study conclusions given that the results prove there is much homogeneity (i.e., significant carceral spending) across the cities included in our sample: the median CRI was calculated to be -0.59 and less than 20% of the sample exhibited spending above parity.

Additionally, the sample size of our study was relatively small, limiting the number of variables in each model. Lastly, our study is limited in its ability to infer causality between each independent variable and the CRI due to the cross-sectional nature of the study design. Despite these limitations, the results of this study provide new insight into the ways in which racial and health inequality shape municipal budgets in major cities across the United States.

Future analyses will examine longitudinal associations between the CRI and direct health outcomes with the goal of establishing causality and motivating effective policy change. We hope to incorporate additional layers of funding at the state and county level to create a fuller picture of investments in each city. In addition, replicating this analysis at the county level could provide a comparison across a state to highlight where categorical funding is lacking and opportunities to shift state investments in counties. The CRI could also be used as a comparison between rural and urban geographies, to draw attention to the possibility that rural areas in a state might be more economically dependent on and utilize carceral systems than most stakeholders are aware of, most of the focus of research on carceral spending so far has largely been on urban areas [32, 42].

## Conclusions

The carceral resource index (CRI) is a new metric that indicates municipal fiscal commitment to carceral systems over health and social systems, beyond commitment to law enforcement alone. Our analyses of the CRI demonstrate that the expansion of carceral systems and the dismantlement of welfare in the United States are deeply intertwined with racial and economic inequality. Continuing to fund carceral systems at the expense of vital disease prevention, treatment, and social support systems will continue to have negative consequences on population health, as evidenced by the response to the COVID-19 pandemic by underfunded public health agencies.

## Supporting information

S1 Fig Residual vs. fitted plots for unadjusted linear models of CRI and ICE variables. (TIF)

S2 Fig Scatter plots of CRI vs. ICE for 50 cities in the United States, fitted with unadjusted polynomial quadratic regression. All ICE variables’ values shown are standardized and scaled by z-transformations.
(TIF)

S3 Fig Residual vs. fitted plots for selected adjusted polynomial quadratic regression models of CRI and ICE variables. All models adjust for proportion of population ≥ 65 yrs of age. Model 1 also adjusts for proportion of population income ≥ $100k, Model 2 adjusts for age, income, and the proportion of population non-Hispanic White.
(TIF)

S4 Fig Normal probability Q-Q plots of adjusted polynomial quadratic regression model between CRI and selected ICE measures. All models adjust for proportion of population ≥ 65 yrs of age. Model 1 also adjusts for proportion of population income ≥ $100k, Model 2 adjusts for age, income, and the proportion of population non-Hispanic White.
(TIF)

S1 Table Adjusted linear regression analysis results for selected model specifications. Adjustments to the linear models 1–4 were equal to those in the polynomial models.
(DOCX)
