# Supplementary figures and images for: Prioritization of carceral spending in U.S. cities: Development of the Carceral Resource Index (CRI) and the role of race and income inequality
Source: PLoS One. 2022 Dec 15;17(12):e0276818. doi: 10.1371/journal.pone.0276818 (PMC9754598; doi:10.1371/journal.pone.0276818)

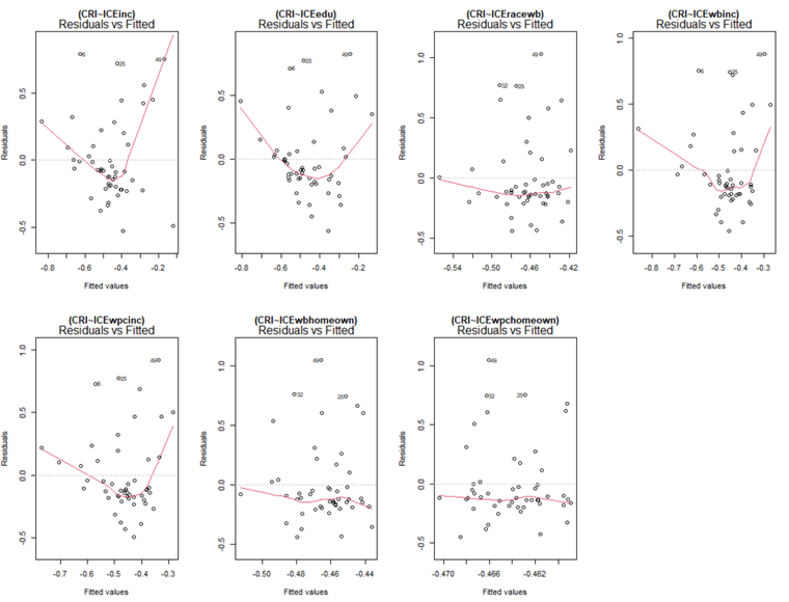

Supplement: S1 Fig — (TIF) [file pone.0276818.s001.tif]

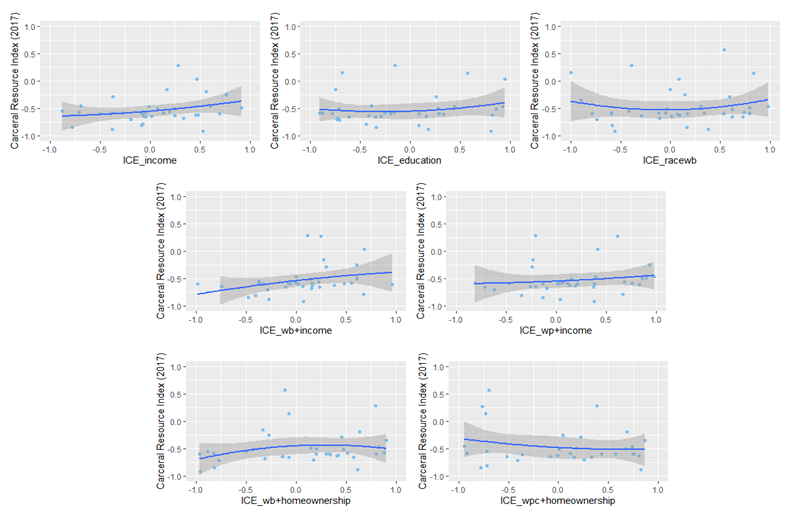

Supplement: S2 Fig — All ICE variables’ values shown are standardized and scaled by z-transformations. (TIF) [file pone.0276818.s002.tif]

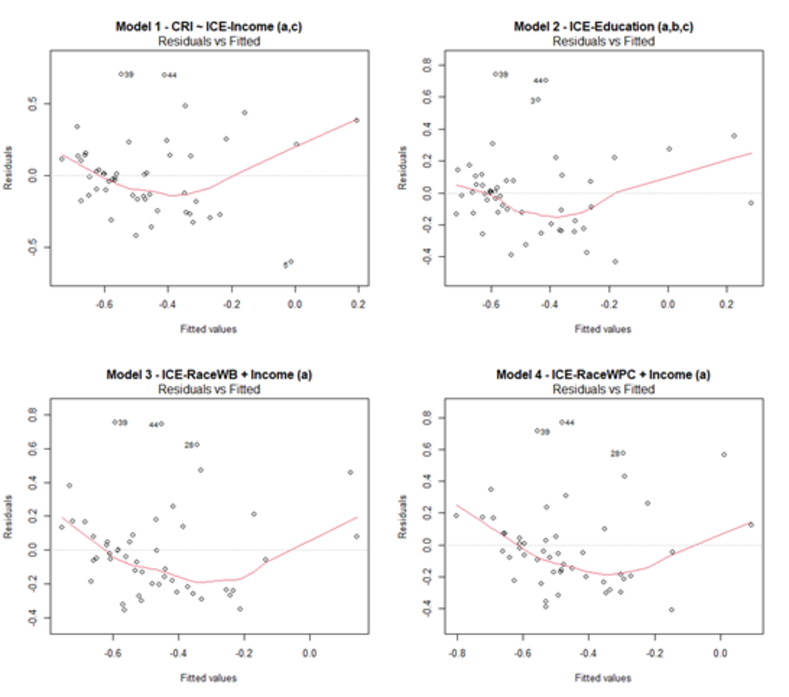

Supplement: S3 Fig — All models adjust for proportion of population ≥ 65 yrs of age. Model 1 also adjusts for proportion of population income ≥ $100k, Model 2 adjusts for age, income, and the proportion of population non-Hispanic White. (TIF) [file pone.0276818.s003.tif]

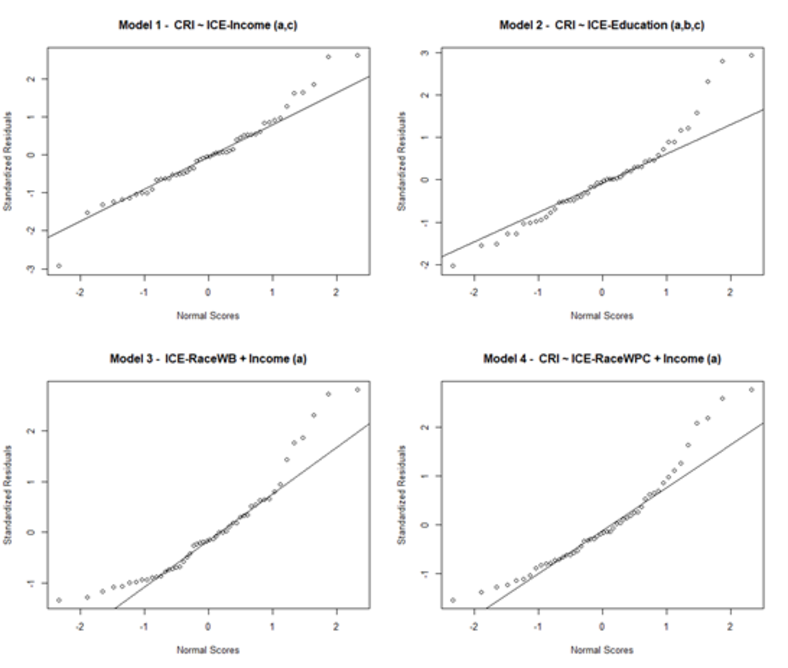

Supplement: S4 Fig — All models adjust for proportion of population ≥ 65 yrs of age. Model 1 also adjusts for proportion of population income ≥ $100k, Model 2 adjusts for age, income, and the proportion of population non-Hispanic White. (TIF) [file pone.0276818.s004.tif]
