# Supplementary material for: Prioritization of carceral spending in U.S. cities: Development of the Carceral Resource Index (CRI) and the role of race and income inequality
Source: PLoS One. 2022 Dec 15;17(12):e0276818. doi: 10.1371/journal.pone.0276818 (PMC9754598; doi:10.1371/journal.pone.0276818)
Supplement: S1 Table — Adjustments to the linear models 1–4 were equal to those in the polynomial models. (DOCX) [file pone.0276818.s005.docx]

| **S1 Table. Adjusted linear regression analysis results for selected model specifications** | | | | | | | | | | | | | | | | | | | | | | | | | | | | |
| --- | --- | --- | --- | --- | --- | --- | --- | --- | --- | --- | --- | --- | --- | --- | --- | --- | --- | --- | --- | --- | --- | --- | --- | --- | --- | --- | --- | --- |
|  | **Model 1** | | | **Model 2** | | | | | | **Model 3** | | | | | | | | | | | **Model 4** | | | | | | | |
| *Predictors* | *Estimates* | *CI* | *p* | | | *Estimates* | *CI* | | | *p* | | *Estimates* | | | | *CI* | | | *p* | | *Estimates* | | | | *CI* | | | *p* |
| (Intercept) | 0.40 | -0.16 – 0.95 | | | 0.155 | -0.07 | -0.83 – 0.70 | | 0.860 | | | | 0.48 | | -0.08 – 1.03 | | | 0.090 | | | | 0.49 | | -0.06 – 1.05 | | | 0.082 | |
| ICEi_ncome_ | 0.08 | -0.00 – 0.17 | | | 0.058 |  |  | |  | | | |  | |  | | |  | | | |  | |  | | |  | |
| ICE_education_ |  |  |  | | -0.05 | -0.18 – 0.08 | | 0.432 | | |  | | |  | | |  | | | |  | |  | | |  | | |
| ICE_wbincome_ |  |  |  | |  |  | |  | | | 0.06 | | | -0.03 – 0.15 | | | 0.162 | | | |  | |  | | |  | | |
| ICE_wpcinc_ |  |  |  | |  |  | |  | | |  | | |  | | |  | | | | 0.05 | | -0.04 – 0.14 | | | 0.246 | | |
| Observations | 50 | | 50 | | | | | | | 50 | | | | | | | | | | 50 | | | | | | | | |
| R^2^ / R^2^ adjusted | 0.306 / 0.276 | | 0.351 / 0.308 | | | | | | | 0.281 / 0.251 | | | | | | | | | | 0.272 / 0.241 | | | | | | | | |
|  | | | | | | | | | | | | | | | | | | | | | | | | | | | | |
